# Editorial: Supporting sustainable behavior change and empowerment in ubiquitous and learning health systems

**DOI:** 10.3389/fdgth.2024.1367567

**Published:** 2024-01-30

**Authors:** Jan David Smeddinck, Rada Hussein, Christopher Bull, Tom Foley, Mark van Gils

**Affiliations:** ^1^ Ludwig Boltzmann Institute for Digital Health and Prevention, Ludwig Boltzmann Gesellschaft, Salzburg, Austria; ^2^ Human-Centered Ubiquitous Media Research Group, LMU Munich, Munich, Germany; ^3^ Open Lab, School of Computing, Newcastle University, Newcastle upon Tyne, United Kingdom; ^4^ Population Health Sciences Institute for Foley, Newcastle University, Newcastle upon Tyne, United Kingdom; ^5^ School of Medicine, University College Dublin, Dublin, Ireland; ^6^ Faculty of Medicine and Health Technology, Tampere University, Tampere, Finland

**Keywords:** digital health, learning health systems, behavior change, health data, health informatics, empowerment

**Editorial on the Research Topic** Supporting sustainable behavior change and empowerment in ubiquitous and learning health systems

## 1 Introduction

The increasing integration of digital health technologies into everyday life marks a transformative shift in healthcare and prevention. Ubiquitous digital health systems include continuous monitoring and intervention capabilities, offering unprecedented opportunities for preventive and rehabilitative healthcare, moving beyond a primary focus on addressing health problems after they symptomatically manifest. This shift is not merely characterized by technological evolution but also by a paradigmatic change towards more personalized, data-driven, and sustainable healthcare approaches. In this editorial, we underscore the potential of ubiquitous and learning health systems to revolutionize healthcare, including fostering sustainable behavior change and empowering individuals in their health journeys.

*Digital health technologies* are increasingly present in our day-to-day lives and are often used continuously, enabling the integration of an increasing variety of data. Such ubiquitous and often mobile digital health systems have considerable potential in enabling long-term—or even life-long disease management. Furthermore, accompanying healthcare monitoring and interventions with a focus on prevention and rehabilitation could reduce the amount and extent of events that require traditional medical treatment. This can lower individual suffering, as well as the burden on public health systems considerably (1, 2) and has the potential to increase quality-adjusted life years (3).

## 2 The promise of learning health systems

In the context of ubiquitous digital health technologies, *learning health systems* have the potential to reshape healthcare by providing continuous, context-aware and reflexively iterated healthcare and prevention systems providing benefits at individual and population levels (4). Longer-term, or even life-long health data, including patient generated and patient contributed data might deliver the concept of a digital health twin (5), which promises to act as the information driving personalized or “precision” health in an increasingly optimized manner. Learning health systems, characterized by their adaptability and iterative improvement, are pivotal in realizing the potential of ubiquitous health technologies given their rapid and continuous development. These systems can leverage data and machine learning as well as learnings from lived and practical experience to evolve and tailor healthcare interventions at the individual and system level with different levels of abstraction of learning loops (6) and with an overarching aim of enhancing the efficacy and efficiency of health services (7). The Free et al., study on pneumonia management demonstrates the potential of data-driven frameworks in supporting clinical decision support. Ultimately, LHS can be a steppingstone towards expanding quality-adjusted life years by promoting proactive health management that considers healthcare and prevention delivery as an inherently iterative process (4) in a complex socio-digital ecosystem [cf. e.g., (8)].

## 3 Challenges, opportunities & the need for sustainable behavior change

The pervasive nature of digital health technologies introduces several challenges, including data security, privacy, and the equitable distribution of healthcare benefits. These challenges necessitate careful consideration and responsible implementation to ensure that these technologies empower rather than disenfranchise individuals (9). Addressing equity in digital healthcare, as discussed in the context of Denmark by Eriksen et al., is crucial to ensure that these technologies benefit all segments of society equally, integrating perspectives on personal and systemic health with a focus on ensuring equitable access, empowerment and emancipation in digital healthcare. A particular challenge exists at the individual level in that *sustainable behavior change* is central to the vision behind approaches to healthcare and prevention that are taking a health-journey accompanying or even life-long approach. Long-term adherence to healthy lifestyle changes, facilitated by digital tools, can significantly improve health outcomes. The EPICURE study by Hayn et al., highlights how digital tools, such as wearables, can support cardiac rehabilitation but also emphasizes the need for larger, randomized controlled trials to fully understand their impact.

## 4 The role of personalization and adaptivity in healthcare

Personalization in healthcare, enabled by data-driven insights and rapidly developing capabilities in machine learning and artificial intelligence (10, 11), is becoming a core ambition of effective digital health systems. It allows for interventions to be tailored to individual needs and preferences, increasing their relevance and effectiveness. E.g., the scalable employment of consumer wearables can support predictive analytics and partially replace traditional manual self-reports, as indicated in the Vuong et al. study on predicting pain ratings in sickle cell disease. Notably, while the technical and procedural building blocks exist for fostering sustainable behavior change in individuals and adaptive improvements in learning health systems, both suffer from a lack of structured investigation. Research approaches that can enable efficient knowledge generation on the complex interplay of variables that determine possible improvements (or deterioration) in individual or population health are clearly needed. This encourages future work that exceeds beyond traditional randomized controlled study designs, enabling the expression (12) and systematic study of complex system configurations and mechanisms for adaptivity. This will require capable and scalable research infrastructures (13), as well as improvements in research methods at scale, for which novel artificial intelligence methods currently offer an exciting avenue of exploration, particularly due to newly evolving capabilities in readily integrating highly varied and sparse multi-modal data streams, as well as converting between structured digital (i.e., “computable”) and unstructured natural language (i.e., easily and efficiently describable) representations.

## 5 Conclusion

This Research Topic brings together relevant articles around sensor-enabled, data-driven, and increasingly ubiquitous digital health. It contributes to the pathway towards individually empowering digital health technology, using e.g., predictive methods that deliver increasingly more precise and preventative approaches, with the larger aim of preserving quality-adjusted life years at the individual and societal level.
